# The Oncogene *MYCN* Modulates Glycolytic and Invasive Genes to Enhance Cell Viability and Migration in Human Retinoblastoma

**DOI:** 10.3390/cancers13205248

**Published:** 2021-10-19

**Authors:** Swatishree Sradhanjali, Padmalochan Rout, Devjyoti Tripathy, Swathi Kaliki, Suryasnata Rath, Rahul Modak, Ruchi Mittal, Tirumala Kumar Chowdary, Mamatha M. Reddy

**Affiliations:** 1 The Operation Eyesight Universal Institute for Eye Cancer, LV Prasad Eye Institute, Bhubaneswar 751024, Odisha, India; swati.gugli@gmail.com (S.S.); pnxrocks@gmail.com (P.R.); 2 School of Biotechnology, KIIT Deemed to Be University, Bhubaneswar 751024, Odisha, India; rahul.modak@kiitbiotech.ac.in; 3 Novo Nordisk, Bangalore 560066, Karnataka, India; 4 Ophthalmic Plastics, Orbit and Ocular Oncology Service, LV Prasad Eye Institute, Bhubaneswar 751024, Odisha, India; drdtripathy@gmail.com (D.T.); suryasnata@lvpei.org (S.R.); 5 The Operation Eyesight Universal Institute for Eye Cancer, LV Prasad Eye Institute, Hyderabad 500034, Telangana, India; kalikiswathi@yahoo.com; 6 Kanupriya Dalmia Ophthalmic Pathology Laboratory, LV Prasad Eye Institute, Bhubaneswar 751024, Odisha, India; dr.rmittal@gmail.com; 7 Department of Pathology, Kalinga Institute of Medical Sciences, Bhubaneswar 751024, Odisha, India; 8 School of Biological Sciences, National Institute of Science Education and Research, Homi Bhabha National Institute, Bhubaneswar 752050, Odisha, India; tkchowdary@niser.ac.in

**Keywords:** retinoblastoma, *MYCN*, shRNA knockdown, microarray analysis, pharmacological inhibition, drug target, metabolism, migration and combination therapy

## Abstract

**Simple Summary:**

Retinoblastoma (RB) is a cancer in children, caused by loss of function of *RB1* gene. Additional factors such as increase in gene copy numbers of oncogenes *MYCN*, *MDM4* and *E2F3* contribute to RB pathogenesis, though their mechanism(s) are not completely understood. We sought to explain the role of *MYCN* in RB pathogenesis. Our data indicate that *MYCN* is overexpressed in RB, and that may contribute to the disease progression by altering the cancer metabolism (glucose metabolism) and cell migration related genes. We also observed that a combination of *MYCN*-inhibition with carboplatin, a drug that is currently used in the treatment of RB has a good synergistic activity against RB. Development of drug-related toxicity and associated long-term side effects is a problem in treatment of RB. *MYCN*-inhibition, in combination with existing drugs, could be a novel, effective therapeutic strategy to reduce high doses of chemotherapy for children that receive prolonged chemotherapy.

**Abstract:**

Retinoblastoma is usually initiated by biallelic *RB1* gene inactivation. In addition, *MYCN* copy number alterations also contribute to RB pathogenesis. However, *MYCN* expression, its role in disease progression and correlation with RB histological risk factors are not well understood. We studied the expression of *MYCN* in enucleated RB patient specimens by immunohistochemistry. *MYCN* is overexpressed in RB compared to control retina. Our microarray gene expression analysis followed by qRT-PCR validation revealed that genes involved in glucose metabolism and migration are significantly downregulated in *MYCN* knockdown cells. Further, targeting *MYCN* in RB cells using small molecule compounds or shRNAs led to decreased cell survival and migration, increased apoptosis and cell cycle arrest, suggesting that *MYCN* inhibition can be a potential therapeutic strategy. We also noted that *MYCN* inhibition results in reduction in glucose uptake, lactate production, ROS levels and gelatinolytic activity of active-MMP9, explaining a possible mechanism of *MYCN* in RB. Taking clues from our findings, we tested a combination treatment of RB cells with carboplatin and *MYCN* inhibitors to find enhanced therapeutic efficacy compared to single drug treatment. Thus, *MYCN* inhibition can be a potential therapeutic strategy in combination with existing chemotherapy drugs to restrict tumor cell growth in RB.

## 1. Introduction

Retinoblastoma (RB) is a pediatric intraocular neoplasm that can either be unilateral or bilateral and accounts for ~4% of total childhood malignancies [1]. RB usually arises due to loss of function of *RB1* tumor suppressor gene present on chromosome 13q14 [2,3]. However, Rushlow et al., 2013 have reported a subset of retinoblastoma patients showing *MYCN* amplifications with no apparent alterations in *RB1* [4]. These *RB1* wildtype and *MYCN* amplified patients can show metastasis, but so far, only intraocular cases were reported. A recent study by Zugbi et al., 2020 showed that the patients with wildtype *RB1* and *MYCN* amplification can metastasize to orbit, and lymph nodes and these patients also showed chemoresistance [5]. *RB1* gene encodes the protein, pRB, which is a key regulator of cell cycle that binds to E2F transcription factors among others and represses cell proliferation in the absence of mitogenic signals [6]. Loss of function of pRB in a susceptible retinal cell initiates benign precursor, retinoma [7]. Alterations in additional pathways contribute further to *RB1* mutations and drive retinoblastoma initiation and progression by activating oncogenes and suppressing tumor suppressors. These changes include copy number alterations and expression levels of *DEK*, *E2F3*, *KIF14*, *MDM4*, *SYK* and *MYCN* [8,9]. Additionally, Afshar et al., 2020 using next generation sequence analysis showed that RB tumor samples can have high frequency of somatic aberrations subsequent to *RB1* deactivation that correlated with high-risk histological features [10].

MYC proteins (c-Myc, MYCL and *MYCN*) are transcriptional regulators that control various cellular processes including cell cycle, cell growth, proliferation, apoptosis and metabolism [11]. The MYC proto-oncoproteins exert their functions through heterodimerizing with its binding partner, MAX, which then binds to E-box sequences present in the promoters of target genes to regulate their expression [12,13]. The expression of MYC proteins is tissue and developmental stage specific. *MYCN* is specifically expressed in neuronal tissues and kidneys [14,15]. Targeted disruption of *MYCN* in mice was found to be embryonically lethal [16]. Deregulation of MYC often results from gross genetic alterations such as copy number changes, chromosomal translocations, increased enhancer activities and dysregulated signal transduction that leads to constitutive overexpression of MYC. The dysregulation of MYC family members has been implicated in a wide variety of cancers [17,18]. For example, *MYCN*, has been shown to be amplified and overexpressed in retinoblastoma [4,19,20,21]. Alterations in copy number or changes in expression of *MYCN* has also been reported in other cancers such as neuroblastoma [22], medulloblastoma [23], glioblastoma multiforme [24,25], Rhabdomyosarcoma [26] and pancreatic tumors [27]. The strategies to target MYC are currently being explored [28]. However, amplification of *MYCN* has been identified since long [18], the mechanisms underlying the role of *MYCN* in RB are just beginning to emerge. There is a need to study whether altered *MYCN* expression in human retinoblastoma samples correlates with any of the high-risk histological factors. Further, it is necessary to identify the genes regulated by *MYCN* that aid in RB progression.

In the current study, we have studied the expression of *MYCN* in retinoblastoma primary tumor tissues and compared it with various histological and clinical parameters. Furthermore, we have explored the possibility of targeting *MYCN* in retinoblastoma using small molecule inhibitors and shRNA approaches. Our data shows that *MYCN* is overexpressed in RB samples both in primarily enucleated as well as chemo-treated tumors. Further, our data confirms that *MYCN* can be targeted using small molecule inhibitors and shRNA approaches. In addition, we have identified the pathways regulated by *MYCN* using gene microarray analysis.

## 2. Materials and Methods

### 2.1. Ethics Approval

This study was accepted by the ethics committee of LV Prasad Eye Institute (protocol number 2019-144-IM-28), Bhubaneswar, India, and followed the tenets of the Declaration of Helsinki.

### 2.2. Cell Culture

Human RB cell lines Y79 and Weri-Rb1 purchased from the American Type Culture Collection (ATCC) (Manassas, VA, USA) were cultured in RPMI-1640 medium (PAN-Biotech, Aidenbach, Germany) containing 10% fetal bovine serum (FBS) (Thermo Fisher Scientific, Waltham, MA, USA) and 1% (*v*/*v*) antibiotics (penicillin-streptomycin-amphotericin B mixture; PAN- Biotech). LRB1 and LRB2 cells derived from bilateral RB patients that were RB1 negative [29] and primary tumor cells isolated from fresh RB tissues were maintained in the same way as the RB cell lines. Human embryonic kidney (HEK) 293T cells (ATCC) were grown in Dulbecco’s Modified Eagle Medium (DMEM; PAN-Biotech) supplemented with 10% FBS. Human retinal pigment epithelium cell line, ARPE-19 (ATCC) was maintained in DMEM: Nutrient Mixture F12 (DMEM/F-12; PAN-Biotech). All the cell lines were verified for the presence of Mycoplasma and were used within 3 months of revival.

### 2.3. Immunoblotting

Total proteins were isolated using RIPA lysis buffer that contained protease (Roche, Basel, Switzerland) and phosphatase inhibitors (Sigma-Aldrich, St. Louis, MO, USA). Bradford reagent (Bio-Rad Laboratories, Hercules, CA, USA) was used to estimate protein quantity. Proteins (equal amounts) were fractionated by sodium dodecyl sulfate polyacrylamide gel electrophoresis (SDS-PAGE) and transferred onto nitrocellulose membrane. The membranes were blocked in 5% bovine serum albumin (BSA, HiMedia laboratories, Mumbai, India) for 1 h followed by overnight incubation at 4 °C with appropriate dilutions of primary antibodies (rabbit anti-*MYCN* monoclonal antibody, 1:1000 dilution, Cell Signaling Technology, Danvers, MA, USA; anti-ß-actin monoclonal antibody, 1:5000 dilution, Sigma-Aldrich; mouse anti-p53 and anti-Bcl2 monoclonal antibodies, 1:500 dilution, Santa Cruz Biotechnology, Dallas, TX, USA). The next day, membranes were washed thrice with Tris-buffered saline with tween 20 (TBS-T) for 5 min each and incubated with respective secondary antibodies for 1 h at room temperature. Following 3 washes in TBS-T, membranes were visualized using chemiluminescence (ECL) reagent (Bio-Rad Laboratories).

### 2.4. Immunohistochemistry

Enucleated RB tissue specimens (*n* = 58; 2013 to 2018) were evaluated for the presence of *MYCN* using immunohistochemistry (IHC). The specimens that did not have significant number of viable tumor cells were excluded from the study. We have included both primarily enucleated and chemotherapy treated eyes for IHC. The enucleated eyes consisted of either group D or E tumors. Formalin fixed paraffin embedded (FFPE) tissue specimens (4 µm) were positioned on to coated glass slides. IHC was performed using the EnVision FLEX Mini Kit (Agilent Technologies, Santa Clara, CA, USA) according to the manufacturer’s instructions. Tissue specimens were immuno-stained with anti-*MYCN* monoclonal antibody at 1:50 dilution and images were acquired using APERIOCS2 slide scanning microscope (Leica Microsystems, Wetzlar, Germany). Images were also captured with a 100X objective on Olympus BX53 microscope (Olympus, Tokyo, Japan) for scoring and data analysis.

Immunoreactivity score was assigned to the tissue specimens showing *MYCN* expression based on the intensity and extent of expression as described previously [30]. Intensity of *MYCN* expression was scored as follows; 0 = negative, 1 = low, 2 = medium and 3 = high. The extent of *MYCN* expression was quantified as the percentage of positively stained cells observed relative to the entire tumor area with a score of 0 for <1%, 1 for 2–10%, 2 for 11–50%, 3 for 51–75% and 4 for >75%. The final immunoreactive score was obtained by multiplying the intensity score by the extent score, with 0 being the minimum score and 12 being the maximum score. An immunoreactive score of >3 was considered positive. The positive samples were further classified into strong, moderate and weak expression. Immunoreactive score of 10 to 12 was considered strong expression; 7 to 9, moderate expression; and 3 to 6, weak expression.

### 2.5. Lentiviral Mediated shRNA Knockdown

*MYCN* was silenced in Y79 and LRB1 cells using two shRNA constructs KD-1 (TRCN0000020695) and KD-2 (TRCN0000020698) (Dharmacon, Lafayette, CO, USA). Lentiviruses containing shRNA constructs that target *MYCN* or scrambled controls were produced in HEK293T cells. Viral supernatant harvested 48 and 72 h post transfection was used for transduction in serum reduced media containing polybrene (4 µg/mL; Sigma-Aldrich).

### 2.6. Determination of Cell Viability

RB cells were treated with either commercially available pharmacological inhibitors: JQ1 (Cayman Chemical Company, Ann Arbor, MI, USA), I-BET762 (APExBIO, Houston, TX, USA), 10058-F4 (Sigma-Aldrich) and 10074-G5 (Sigma-Aldrich) or inhibited by shRNA mediated approach. Cells were seeded at a density of 0.1 × 10^6^ cells/mL and treated for 48 h with small molecule inhibitors. Cell viability was determined by trypan blue dye (Sigma-Aldrich) exclusion assay. The reduction in cell viability was measured following inhibitor treatment or shRNA-mediated knockdown and compared to untreated or scrambled controls.

### 2.7. Apoptosis Assay

RB cells (1 × 10^6^ cells) were treated with inhibitors for 48 h and stained with Annexin V and Propidium Iodide (PI) as per manufacturer’s instructions (Miltenyi Biotec, Bergisch Gladbach, Germany). Stained cells were immediately analyzed on a cytoFLEX flow cytometer (Beckman Coulter Life Sciences, Brea, CA, USA).

### 2.8. Cell Cycle Analysis

The changes in cell-cycle distribution upon *MYCN* inhibition were determined by analyzing the DNA content by flow cytometry using PI (Sigma-Aldrich) staining. Briefly, 1 × 10^6^ cells were washed twice with Dulbecco’s phosphate buffered saline (DPBS; PAN-Biotech) and fixed in 70% ice cold ethanol overnight. After ethanol fixation, cells were washed twice with DPBS and treated with 50 µg/mL of RNase-A (QIAGEN, Hilden, Germany) and incubated at 37 °C for 30 min. Prior to flow cytometry analysis, cells were washed with DPBS and stained with 50 µg/mL of PI.

### 2.9. Glucose Uptake

Fluorescent glucose analog 2-(*N*-(7-nitrobenz-2-oxa-1,3-diazol-4-yl) amino)-2-deoxyglucose (2-NBDG); (Thermofisher Scientific) was used to measure glucose uptake by flow cytometry. Cells (1 × 10^6^) were incubated in 30 µM of 2-NBDG in PBS for 20 min at 37 °C, afterwards washed in cold DPBS twice and subjected to flow cytometric evaluation. Alteration in glucose uptake post 48 h of inhibitor treatment was compared relative to control cells.

### 2.10. Lactate Assay

The lactate content was measured using the commercially available lactate assay kit (BioVision, Mountain View, CA, USA) as per the manufacturer’s directions. Cells (1 × 10^6^) were incubated for 4 h in serum free medium prior to colorimetric analysis and relative changes in lactate levels were determined in inhibitor treated and shRNA knockdown samples compared to untreated or scrambled controls.

### 2.11. Measurement of Reactive Oxygen Species (ROS)

The relative intracellular ROS levels were determined by using 2′,7′-dichlorofluorescein diacetate (DCF-DA; Merck, Darmstadt, Germany). Cells (1 × 10^6^) were incubated with DCF-DA (20 µM) in phosphate-buffered saline (PBS) for 5 min at 37 °C, followed by gentle washing with ice cold PBS twice and analyzed using flow cytometry.

### 2.12. Drug Combination Assay

Drug synergy analysis was performed based on Chou and Talalay’s method [31]. Cells were treated with carboplatin and *MYCN* inhibitors either alone or in combination for 48 h. IC50 of individual drugs were calculated and drug concentrations below and above IC50 were taken for the combination assay. Cell viability was determined using trypan blue assay. Percentage of cell growth was determined in treated samples relative to control. Fraction affected (Fa) was calculated using a formula, Fa = 1 − (% growth/100). Fa values were calculated for each concentration of single drug (JQ1, 10058-F4 or carboplatin) and the combinations. Combination indices (CI) were determined for all the drug combinations tested. CI value less than 1 is considered synergistic, equal to 1 additive and more than 1 antagonistic [31].

### 2.13. Wound Healing Assay

Approximately 2 × 10^6^ cells were seeded onto 6-well poly-d-lysine (0.01%) coated plates, serum starved and maintained in RPMI-1640 containing 1% Penicillin/Streptomycin/Amphotericin-B mix. After 24 h, two fine scratches (perpendicular to each other) were made using 200 µL micropipette tip. Cell culture media was removed, and cells were washed gently with sterile DPBS. Fresh RPMI-1640 medium supplemented with 2% FBS and 1% Penicillin/Streptomycin/Amphotericin-B mixture was added. Microscopic pictures of the wound created were recorded at 0- and 24-hours’ time interval in scrambled and *MYCN* knockdown cells.

### 2.14. Microarray Analysis

Microarray analysis was performed to evaluate the gene expression profile in scrambled and *MYCN* knockdown Y79 cells. The microarray platform Human GXP 8X60k was procured from Agilent Technologies. Scrambled, *MYCN* KD-1 and *MYCN* KD-2 were subjected to microarray analysis.

#### 2.14.1. Data Interpretation and Identification of Differentially Expressed Genes (DEGs)

The normalization was carried out using GeneSpring GX 14.5 Software. Percentile Shift Normalization method was used for normalizing the data. Analysis was carried out with respect to control samples. log_2_FC (fold change) > 1 or <−1 were considered significantly different between the scrambled control group and the *MYCN* knockdown group.

#### 2.14.2. Gene Ontology (GO), Pathway and Function Enrichment Analysis of DEGs

To determine the GO function of the prioritized genes as well as their significantly enriched pathways, GO function and Kyoto Encyclopedia of Genes and Genomes (KEGG) pathway analysis of DEGs were performed using DAVID (The Database for Annotation, Visualization and Integrated Discovery) (http://david.abcc.ncifcrf.gov/) accessed on 14 October 2021. *p*-value < 0.05 was considered statistically significant.

### 2.15. RNA Isolation and Validation of Identified Genes

Total RNA from cell lines or tissues was extracted using Trizol reagent (Thermo Fisher Scientific). RNA was quantified using a Biospectrophotometer (Eppendorf, Hamburg, Germany). RNA was reverse transcribed using cDNA synthesis kit (Bio-Rad Laboratories). Real time quantitative polymerase chain reaction (qRT-PCR) analysis was performed using gene specific primers and SYBR green master mix (Bio-Rad Laboratories). Beta 2 microglobulin (ß2M) was used as an endogenous control.

### 2.16. Gelatin Zymography

Gelatinolytic activity of MMP9 and MMP2 upon *MYCN* inhibition in Y79 and LRB1 cells was determined by substrate gelatin zymography. Equal amount of proteins were separated on 10% SDS-PAGE gels containing 0.1% gelatin (Amresco, Solon, OH, USA). The gels were washed thrice for 15 min each in 2.5% Triton X-100 washing buffer and then incubated in incubation buffer containing 50 mM Tris-HCl, 10 mM CaCl_2_, 1 M ZnCl_2_ and 200 mM NaCl, pH 7.5 at 37 °C for 18 to 20 h. Subsequently, gels were stained with Coomassie solution (0.05% Coomassie brilliant blue R-250, in 40% methanol and 10% acetic acid) and destained with destaining solution (20% methanol and 10% acetic acid) to visualize the clear zones of gelatinolytic activity of MMPs against the blue background.

### 2.17. Statistical Analysis

Data were presented as the mean ± SEM and statistically significant differences were identified with Student’s *t* test as indicated in the figure legends.

## 3. Results

### 3.1. Overexpression of *MYCN* in Cell Lines and Enucleated Patient Specimens of Human Retinoblastoma

**Figure 1:**
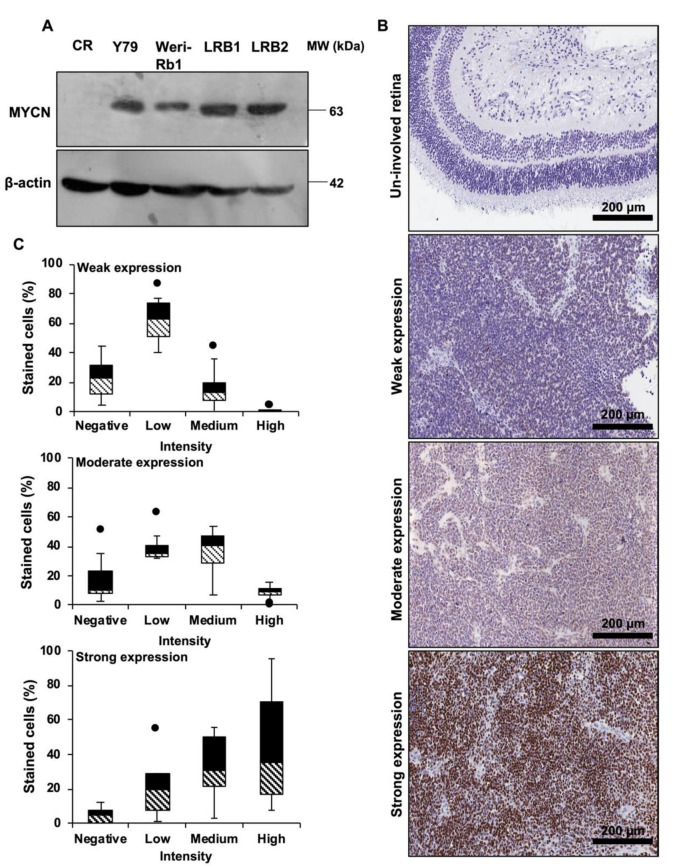
*MYCN* is highly expressed in RB. (**A**) Immunoblot showing overexpression of *MYCN* in RB cells compared to control retina (CR). (**B**) Illustrative Immunohistochemical images of *MYCN* expression in un-involved retina and RB patient specimens showing weak, moderate and strong expression. (**C**) Box plots depicting the distribution of cells based on intensity scoring of *MYCN* expression.

*MYCN* protein expression in human RB cell lines Y79 and Weri-Rb1 and patient derived cells LRB1 and LRB2 was determined by immunoblotting and compared it to control retina. Elevated expression of *MYCN* protein was observed in human RB cell lines (Figure 1A, Full blot in Figure S6). However, Weri-RB1 had relatively lower expression of *MYCN* compared to Y79. This could be due to gain of *MYCN* in Weri-RB1 and amplification in Y79 [32]. LRB1 and Y79 cells had similar expression of *MYCN* and these two cell lines were used in further experiments. Next, the expression level of *MYCN* protein was evaluated in archived RB tissue specimens (*n* = 58) by immunohistochemical staining and positive staining for *MYCN* was observed in 36 samples (62.06%). The scoring of IHC slides based on the intensity and extent of positive expression was carried out as described previously [30]. Among the 36 positively stained samples, weak, moderate and strong expression of *MYCN* was observed in 14 (38.88%), 13 (36.11%) and 9 (25%) specimens, respectively. No expression of *MYCN* protein was observed in the adjacent conserved retina. The typical microscopic images of the stained tissue samples (Figure 1B) with their respective distribution of positively stained cells based on intensity score are shown (Figure 1C). The extent of *MYCN* expression was correlated with demographical parameters and histological risk factors such as optic nerve, choroidal, anterior chamber and scleral invasion and no statistically significant association was found (Table 1).

### 3.2. Pharmacological Inhibition of *MYCN* Led to Decreased RB Cell Viability

**Figure 2:**
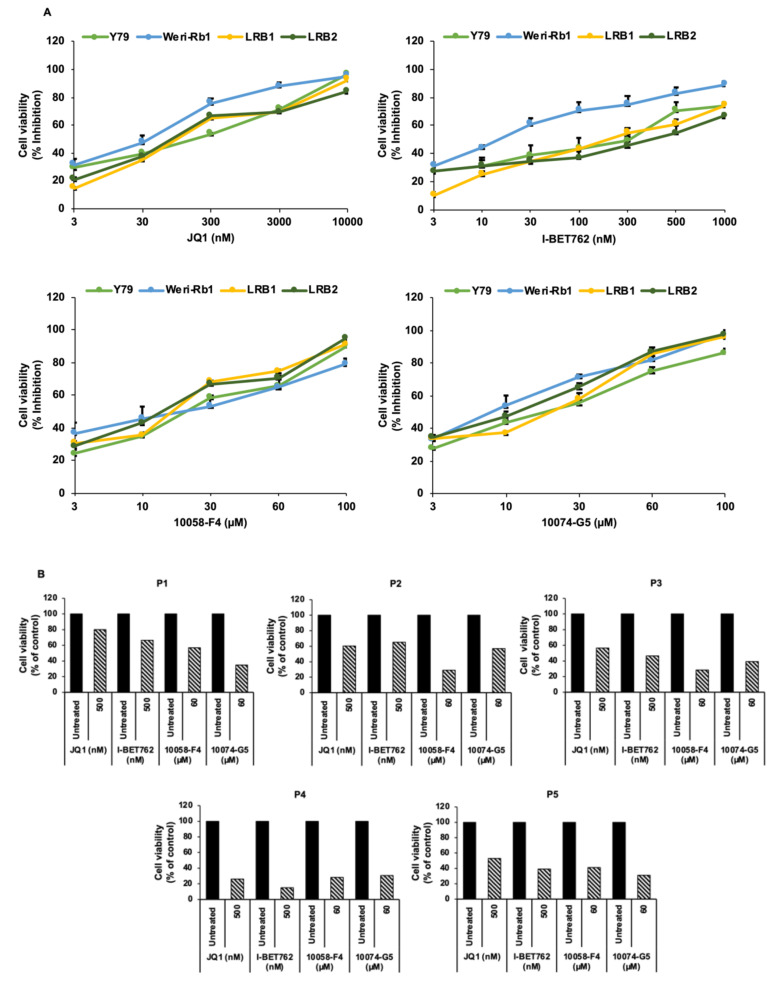
Pharmacological inhibition of *MYCN* using small molecule inhibitors. (**A**) Cell viability of RB cell lines; Y79 and Weri-Rb1 and patient derived cells; LRB1 and LRB2 upon treatment with small molecule inhibitors. The error bars represent standard error of mean (SEM). (**B**) Cell viability of primary tumor cells (P1-P5) after treatment with *MYCN* inhibitors.

We next investigated the inhibition of *MYCN* as a therapeutic strategy in RB cells by using small molecule inhibitors. We have employed inhibitors based on two different mechanisms of action; 1. bromodomain inhibitors, JQ1 and I-BET762, and 2. inhibitors of MYC-MAX interaction, 10058-F4 and 10074-G5. The RB cell lines Y79 and Weri-Rb1 and patient derived cells LRB1 and LRB2 were treated with the above-mentioned inhibitors and cell viability was measured. Upon *MYCN* inhibition, a significant reduction in cell survival was observed in RB cells in a concentration dependent manner in comparison to untreated controls (Figure 2A). The IC_50_ values of all the pharmacological inhibitors for each cell line were determined and are shown in Table S1. Similarly, the changes in cell viability in response to *MYCN* inhibition were verified on primary RB patient samples (n = 5) that showed *MYCN* expression and found to show reduction in cell viability in similar manner as cell lines (Figure 2B). As the tumor sample available was limited, we could not test many concentrations. We selected drug concentration for each individual drug at which, we observed around 70–80% reduction in cell viability in cell lines. Further, to investigate the effects of *MYCN* inhibitors on untransformed cells, we treated retinal pigment epithelial cells (ARPE-19) with concentrations of each drug effective against RB cells and found that *MYCN* inhibition did not significantly change the viability of ARPE-19 cells (Figure S1).

### 3.3. *MYCN* Inhibition Induces Cell Cycle Arrest at G0/G1 Phase and Triggers Apoptosis in RB Cells

**Figure 3:**
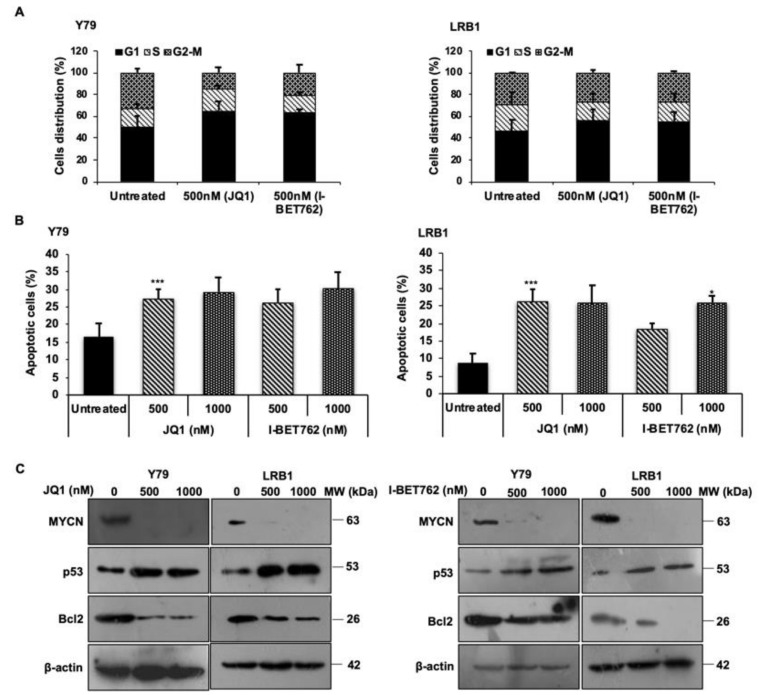
*MYCN* inhibition promotes cell cycle arrest at G0/G1 phase and induces apoptosis in RB cells. (**A**) Fraction of cells accumulated in each phase of the cell cycle after treatment with JQ1 and I-BET762 in Y79 and LRB1 cells. (**B**) Total apoptosis in Y79 and LRB1 cells treated with JQ1 and I-BET762. (**C**) Protein expression levels of *MYCN*, Bcl-2 and p53 upon inhibition with JQ1 and I-BET762 in Y79 and LRB1 cells. The error bars represent the standard error of mean (* *p* < 0.05 vs. untreated, *** *p* < 0.001 vs. untreated).

To evaluate the effects of *MYCN* inhibition on RB cell cycle progression, we performed cell cycle analysis using PI staining. Y79 and LRB1 cells treated with JQ1 and I-BET762 were subjected to flow cytometry evaluation. It was found that cells were accumulated in G0/G1 phase of the cell cycle as shown in Figure 3A. Similar results were obtained in Y79 and LRB1 cells treated with 10058-F4 and 10074-G5 (Figure S2A). Further, *MYCN* inhibition also resulted in increased apoptosis in Y79 and LRB1 cells compared to untreated control cells (Figure 3B and Figure S2B). The protein levels of *MYCN* were measured by immunoblotting and the results showed that *MYCN* levels were decreased in response to treatment with JQ1 and I-BET762 inhibitors (Figure 3C, full blots in Figure S6). Additionally, the levels of p53 and Bcl2 proteins were evaluated in Y79 and LRB1 cells treated with JQ1 and I-BET762 inhibitors. The data showed a diminished expression of Bcl2 protein and an elevated expression of p53 protein upon *MYCN* inhibition (Figure 3C, full blots in Figure S6), which further corroborates that *MYCN* inhibition triggers apoptosis possibly via the induction of p53 mediated apoptotic pathway in RB.

### 3.4. shRNA Mediated Knockdown of *MYCN* Inhibits Cell Proliferation in RB

**Figure 4:**
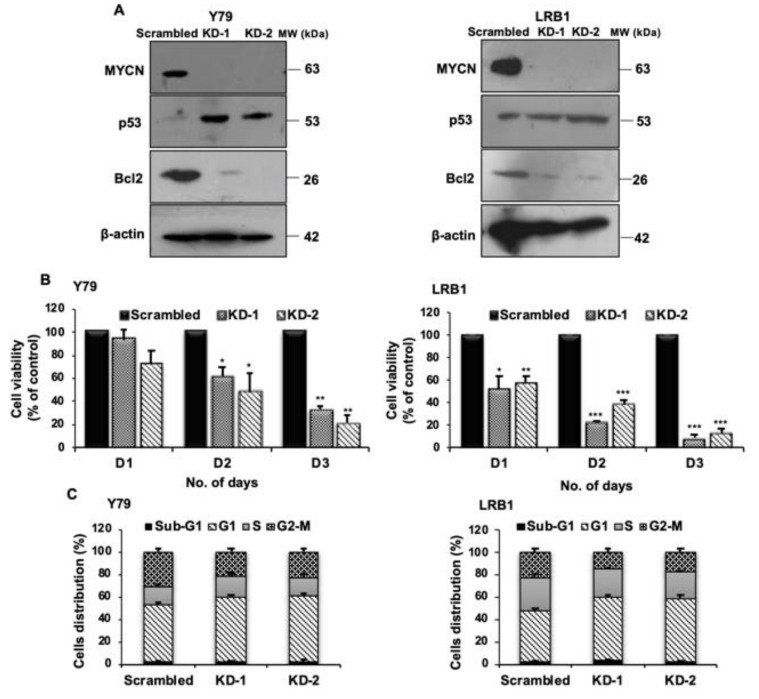
*MYCN* knockdown in RB cells results in decreased cell viability and cell cycle arrest at G0/G1 phase. (**A**) Confirmation of *MYCN* knockdown and the levels of p53 and Bcl-2 proteins upon *MYCN* knockdown in Y79 and LRB1 cells. (**B**) Effect of *MYCN* knockdown on RB cell viability in comparison with the scrambled control cells. (**C**) Cell cycle analysis in response to *MYCN* knockdown. The error bars represent standard error of mean (* *p* < 0.05, ** *p* < 0.01, *** *p* < 0.001 determined by Student’s *t*-test).

Next, to confirm the role of *MYCN* in RB, *MYCN* was targeted using shRNA mediated lentiviral knockdown in Y79 and LRB1 cells. Two shRNA constructs (KD-1 and KD-2) targeting *MYCN* were used in the study. Cells transduced with scrambled shRNA served as control. *MYCN* knockdown was confirmed by immunoblotting (Figure 4A, full blots in Figure S6). The effects of *MYCN* knockdown on cell viability of Y79 and LRB1 cells were determined up to 3 days. A significant decrease in cell viability was observed in Y79 cells on day 2 and day 3. LRB1 cells were more sensitive to shRNA knockdown compared to Y79 cells (Figure 4B). Further, cell cycle analysis was performed, and the results showed accumulation of cells in G0/G1 phase (Figure 4C) suggesting the role of *MYCN* in RB cell growth and proliferation. Additionally, the levels of pro-apoptotic p53 and anti-apoptotic Bcl2 proteins were assessed upon *MYCN* knockdown in RB cells. An elevated expression of p53 and reduced expression of Bcl2 was observed (Figure 4A, full blots in Figure S6).

### 3.5. Microarray Analysis upon *MYCN* Knockdown

To understand the molecular functions and pathways regulated by *MYCN* in RB, we performed microarray analysis of Y79 cells with *MYCN* knockdown and scrambled controls. The expression of various genes was profiled using Agilent’s microarray platform Human GXP 8X60k. The dataset was submitted in the repository of “Gene Expression Omnibus” with the accession number GSE168903, https://www.ncbi.nlm.nih.gov/geo/query/acc.cgi?acc=GSE168903, accessed on 14 October 2021.

#### 3.5.1. Identification of Differentially Expressed Genes (DEGs), Gene Ontology (GO), Enriched Functions and Pathway Analysis

**Figure 5:**
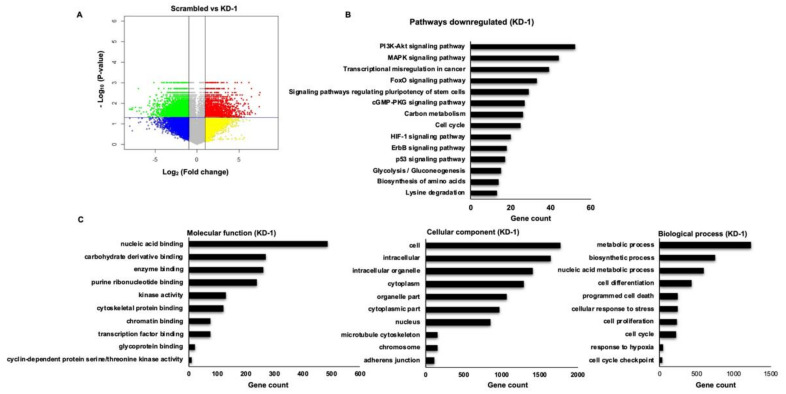
Microarray analysis of differentially expressed genes (DEGs). (**A**) Volcano plot constructed using fold change and *p*-values to compare the gene expression changes between scrambled and *MYCN* knockdown (KD-1) Y79 cells. The green spots indicate significantly downregulated genes with log_2_ (fold change) < −1 and *p*-value < 0.05, and red spots represent the significantly upregulated genes with log_2_ (fold change) > 1 and *p*-value < 0.05. the yellow and blue spots indicate the non-significant changes in gene expression. (**B**) KEGG pathway enrichment analysis of downregulated DEGs upon *MYCN* knockdown. (**C**) GO functional enrichment analysis of downregulated DEGs in response to *MYCN* knockdown. GO terms are enriched into three subgroups: Molecular function, Cellular component and Biological process. KEGG: Kyoto Encyclopedia of Genes and Genomes, GO: Gene Ontology.

Significant genes upregulated with fold change ≥ 1 (logbase2) and downregulated with fold change ≤ −1 (logbase2) in the *MYCN* knockdown samples with respect to scrambled controls were identified. There were 12,642 mRNAs considered to be differentially expressed between scrambled and KD-1 group. Among the differential mRNAs, 7388 were downregulated and 5254 were upregulated (Figure 5A). Similarly, 11,731 mRNAs were found to be differentially expressed between scrambled and KD-2 group, of which, 6601 mRNAs were downregulated and 5130 mRNAs were upregulated (Figure S3A).

To investigate the functions of the identified DEGs, gene enrichment analysis was performed by online software tool DAVID. Next, KEGG pathway analysis was performed to identify potential pathways regulated by the DEGs in RB. A total of 14 significantly enriched pathways for downregulated DEGs were identified and represented graphically (Figure 5B and Figure S3B). The downregulated DEGs were further classified into sub-functional groups including molecular function (MF), cellular component (CC) and biological process (BP). The top 10 significantly enriched GO terms in each of the functional groups were identified (Figure 5C and Figure S3C).

#### 3.5.2. Screening of the Identified DEGs Related to Glucose Metabolism and Migration

Among the significantly enriched pathways of the DEGs identified by microarray analysis, our study was focused mainly on two important pathways: glucose metabolic pathway and migration.

##### Validation of Identified Metabolic Genes

**Figure 6:**
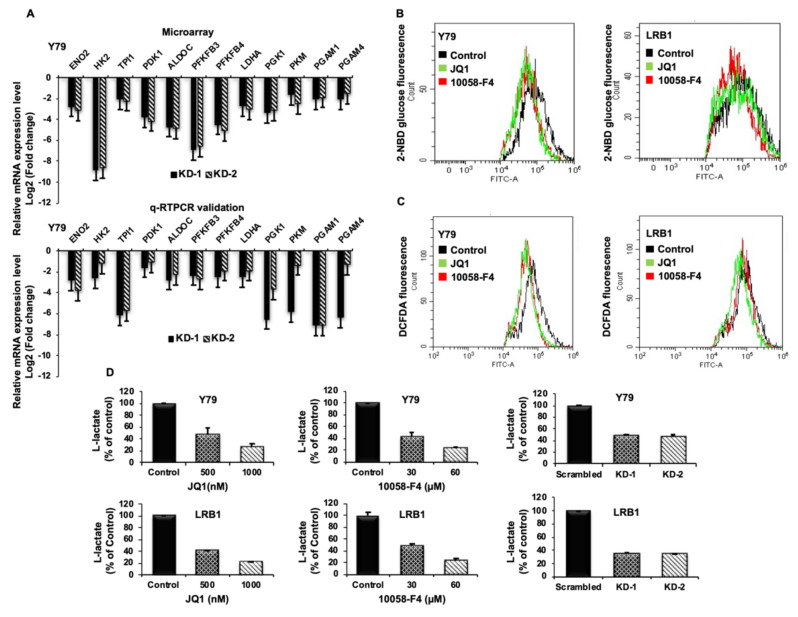
The impact of *MYCN* inhibition on metabolic parameters in RB cells. (**A**) Top 12 downregulated differentially expressed metabolic genes identified by microarray data analysis (upper panel) and qRT-PCR validation of the identified DEGs (lower panel). (**B**,**C**) Decreased levels of glucose uptake and reactive oxygen species (ROS) were observed upon *MYCN* inhibition compared to the untreated control cells. (**D**) *MYCN* inhibition with small molecule inhibitors and shRNA knockdown led to decreased lactate levels in Y79 and LRB1 cells. The error bars represent standard error of mean.

As altered metabolism is one of the hallmarks of cancers, we specifically screened for significantly downregulated genes involved in glucose metabolism upon *MYCN* knockdown. Enolase 2 (ENO2), Hexokinase 2 (HK2), Trios phosphate isomerase 1 (TPI1), Pyruvate dehydrogenase kinase 1 (PDK1), Aldolase C (ALDOC), 6-phosphofructo-2-kinase/fructose-2,6-biphosphatase 3 (PFKFB3), 6-phosphofructo-2-kinase/fructose-2,6-biphosphatase 4 (PFKFB4), Lactate Dehydrogenase A (LDHA), Phosphoglycerate kinase 1 (PGK1), Pyruvate kinase M (PKM), Phosphoglycerate mutase 1 (PGAM1) and Phosphoglycerate mutase 4 (PGAM4) were among the top 12 candidate genes that were found to be downregulated upon *MYCN* knockdown. To confirm the data obtained from microarray analysis, we validated the mRNA expression of the above-mentioned candidate genes by qRT-PCR using gene specific primers (Table S2). The expression of the validated genes in cells with *MYCN* knockdown compared to scrambled controls was shown to be in agreement with the microarray analysis in Y79 (Figure 6A) and LRB1 cells (Figure S4A). Further, we also verified the expression of DEGs in inhibitor treated Y79 and LRB1 cells (Figure S4B).

##### Effect of *MYCN* Inhibition on Metabolic Parameters

To verify the regulation of *MYCN* on metabolic functions, we evaluated the effects of *MYCN* inhibition on metabolic parameters such as glucose uptake, lactate production and intracellular ROS. Cells were treated with JQ1 and 10058-F4 for 48 h, stained with 2-NBDG and analyzed by flow cytometry. RB cells showed a significant decrease in glucose uptake levels upon treatment with inhibitors. (Figure 6B). In addition, *MYCN* inhibition led to decreased ROS levels in RB cells compared to the untreated control cells (Figure 6C). Further, a significant decrease in L-lactate levels was observed in Y79 and LRB1 cells treated with JQ1 and 10058-F4 as well as in *MYCN* knockdown cells relative to the untreated/scrambled controls (Figure 6D).

##### *MYCN* Regulates Genes Involved in Cellular Migration and Metastasis

**Figure 7:**
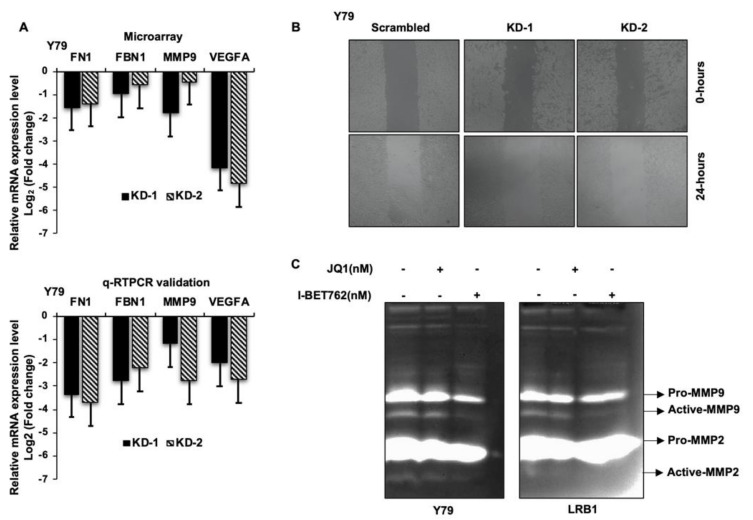
The effects of *MYCN* inhibition on cell migration in RB cells. (**A**) The bar graphs represent the top 4 downregulated genes involved in cellular migration and metastasis, upon *MYCN* knockdown in Y79 cells identified by microarray analysis (upper panel) and qRT-PCR validation of the identified genes (lower panel). (**B**) Reduced migration of cells was observed upon *MYCN* knockdown compared to the scrambled control cells. (**C**) Decreased gelatinolytic activity of active-MMP9 was observed in RB cells following *MYCN* inhibition. The error bars represent the standard error of mean.

It is evident from the existing literature that extracellular matrix (ECM) undergoes substantial remodeling during cancer progression and performs a key role in cancer metastasis. Decreased mRNA levels of fibrillin (FBN1), fibronectin (FN1), matrix-metalloproteinase 9 (MMP9) and vascular endothelial growth factor A (VEGFA) were identified in our microarray data analysis, and we have further validated their mRNA expression in RB cells in response to *MYCN* knockdown. qRT-PCR revealed a decreased expression of FBN1, FN1, MMP9 and VEGFA mRNA compared to the scrambled cells (Figure 7A). Next, to ascertain the effects of *MYCN* inhibition on RB cell migration, wound healing assay was performed in Y79 with *MYCN* knockdown. A decreased cellular migration was observed in *MYCN* knockdown cells compared to scrambled controls (Figure 7B). Additionally, the activity of MMP9 was determined by gelatin zymography and a decrease in gelatinolytic activity of active-MMP9 was observed in Y79 and LRB1 cells treated with JQ1 and I-BET762 compared to the untreated cells (Figure 7C). Overall, our data suggests that targeting *MYCN* may limit tumor cell migration in RB possibly via downregulating the expression of ECM proteins and VEGFA.

### 3.6. Drug Combination Effects of *MYCN* Inhibitors and Carboplatin on Retinoblastoma Cell Viability

**Figure 8:**
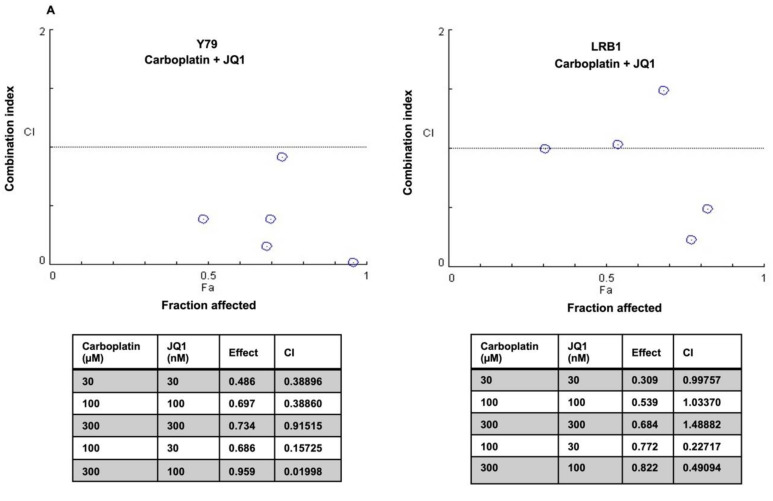

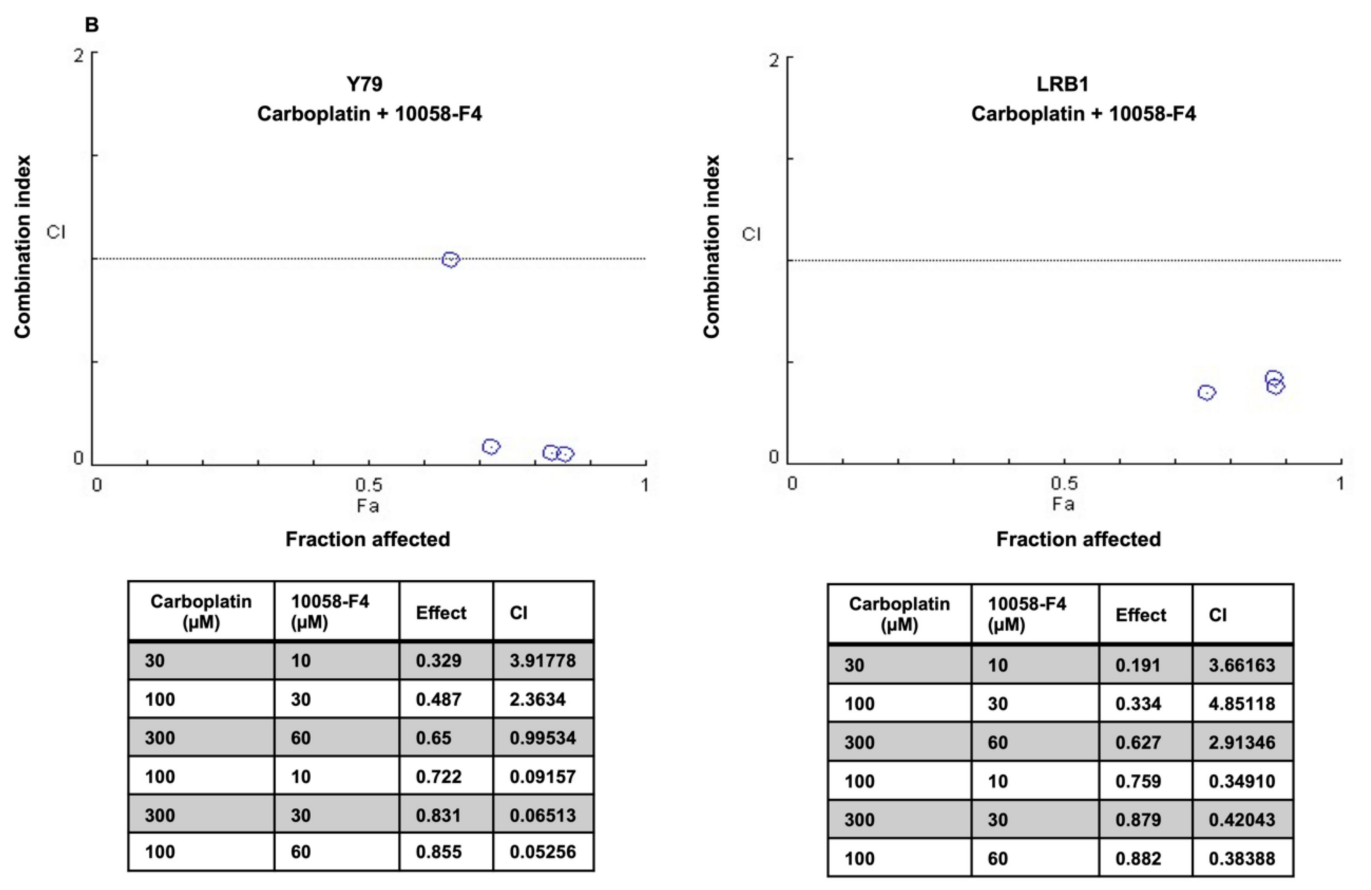
Combination effects of carboplatin and small molecule inhibitors of *MYCN* on RB cell viability. Y79 and LRB1 cells were treated with carboplatin, JQ1 and 10058-F4 either alone or in combination. Combination Indices (CI) were determined. CI < 1 was considered synergistic, CI = 1 as additive and CI > 1 as antagonistic. (**A**) A profound growth inhibitory effect of carboplatin and JQ1 combination was observed in Y79 cells for all the combinations tested, exhibiting synergism whereas variable effects were observed in LRB1 cells. (**B**) Combination treatment of carboplatin and 10058-F4 resulted in synergism as well as antagonism compared to the single drug treatment.

Recurrent retinoblastoma is a therapeutic challenge in a subset of RB patients and some children also show poor response to chemotherapy. The efficacy of chemotherapy drugs can be enhanced by combining them with other therapeutic molecules. In this study, we have tested the drug combination effects of carboplatin and small molecule inhibitors of *MYCN* on RB cell viability in vitro. RB cell lines (Y79 and LRB1) were treated with JQ1, 10058-F4 and carboplatin either alone or in combination. The data were analyzed by median effect combination index (CI) method and CI values were plotted against the values of fraction affected (Fa). The CI values < 1 was considered synergistic, CI = 1, additive and CI > 1, antagonistic. The data indicated that Y79 and LRB1 cells responded differently to the combination therapy. A profound growth inhibitory effect of carboplatin and JQ1 combination was observed in Y79 cells, and they were sensitive to all the concentrations of drug combinations tested, exhibiting synergism (Figure 8A). However, combination of carboplatin and JQ1 yielded variable effects in LRB1 cells showing synergism in two of the drug combinations, (100 µM carboplatin and 30 nM JQ1) and (300 µM carboplatin and 100 nM JQ1) and presented additive effects in two combinations and antagonistic effect in one combination (Figure 8A). Likewise, combination treatment of Y79 and LRB1 cells with carboplatin and 10058-F4 resulted in both enhanced inhibitory effects (synergism) as well as adverse effects (antagonism) on cell growth compared to the single drug treatment (Figure 8B). Dose response curves were also plotted for individual drugs as well as combination of carboplatin and JQ1 or 10058-F4 (Figure S5). Even though drug synergism was observed in RB cells, the mechanistic insights into the synergistic activities of carboplatin and *MYCN* inhibitors are yet to be deciphered and should be considered for future studies. Our work provides a rationale to develop targeted combination therapy for subgroups of RB patients with *MYCN* overexpression and chemoresistance.

## 4. Discussion

The inactivation of both the alleles of RB1 initiates benign lesion, retinoma in the retina of the susceptible eye [7]. Loss of RB1 accompanied by gradual increase in genomic instability in the susceptible retinal cells drives disease progression from non-proliferative retinoma to malignant retinoblastoma. Changes in gene copy number and expression of oncogenic transcription factor, *MYCN* along with other oncogenes such as *MDM4*, *KIF14*, *E2F3*, *SYK* and *DEK* have been reported in RB [8], reviewed in [33]. *MYCN* expression was shown to be inherently high in differentiating cone precursor cells that are thought to be the cell of origin for RB and further, RB cells are dependent on *MYCN* expression for their survival and proliferation [34]. Even though, *MYCN* has been shown to be amplified in RB, its amplification did not correlate with any of the histological high-risk factors such as optic nerve or choroidal invasion [35]. However, in contrast with RB, neuroblastoma, another childhood malignancy, with *MYCN* amplification was shown to correlate with poor prognosis [22,36]. Consequently, we evaluated the expression level of *MYCN* in RB patient specimens to corroborate if *MYCN* expression correlated with histological high-risk factors. *MYCN* overexpression was seen in 62% of the RB patient specimens compared to the adjoining healthy looking uninvolved retina and the expression did not significantly correlate with advanced disease features. The lack of *MYCN* positive staining in the remaining samples could be due to the short half-life of *MYCN* protein or a subset of samples do not have *MYCN* expression. Nonetheless, in the present study, the *MYCN* expression data might also reflect the mouse model data wherein the authors have demonstrated that *MYCN* expression along with RB1 deficiency is required for initial tumor formation, however, progression and maintenance of RB is independent of *MYCN* expression [37]. Based on our findings and study by Wu et al.; 2017, we speculate that *MYCN* plays a role in early events of RB tumorigenesis. However, histological evaluation of disease progression is not feasible as incisional biopsy incurs the risk of metastasis in RB in contrast with other tumors.

*MYCN* plays a central role in regulating a spectrum of cellular functions that drives the oncogenic processes, which include cell cycle, cell growth, proliferation, apoptosis and metabolism [18]. Even though targeting *MYCN* can be a robust strategy for the treatment of various *MYCN*-driven tumors, direct inhibition of *MYCN* has been challenging due to its undruggable structural conformation [28]. Indirect targeting of *MYCN* using small molecule inhibitors such as bromodomain inhibitors and inhibitors that disrupt the interaction between MYC and its binding partner, MAX has been explored in different cancers [38,39,40,41,42]. In the present study, we targeted *MYCN* pharmacologically in RB cells using two classes of small molecule inhibitors: selective bromodomain inhibitors and inhibitors of MYC-MAX interaction, and by shRNA mediated knockdown approach. *MYCN* inhibition resulted in a significant decrease in RB cell survival compared to the untreated or scrambled controls. Additionally, we tested the inhibitory effects of these small molecule inhibitors on *MYCN* overexpressing primary RB patient specimens, and untransformed retinal pigment epithelial cells (ARPE-19). A decreased cell viability in response to *MYCN* inhibition was observed in RB patient specimens in a similar manner as cell lines, whereas no significant inhibition was detected in ARPE-19 cells. Assessing the inhibitory effects of *MYCN* on primary cells is particularly important as no animal models that truly mimic human RB are available so far. Further, *MYCN* inhibition led to increased apoptosis and cell cycle arrest at G0/G1 phase. Increased expression of p53 and decreased expression of Bcl2 proteins upon *MYCN* inhibition was also observed in RB cells. Collectively, our data demonstrates that targeting *MYCN* inhibits cell growth, induces cell cycle arrest and promotes apoptosis in RB cells.

To further explore and identify the regulatory networks downstream of *MYCN* in RB, we performed DNA microarray analysis in scrambled and *MYCN* knockdown cells. The KEGG functional enrichment analysis of identified downregulated DEGs showed that they were mainly enriched in the cancer related pathways such as PI3K-Akt signaling, MAPK signaling, transcriptional misregulation in cancer, FoxO signaling, carbon metabolism, glycolysis and p53 signaling among others. To characterize the biological function of the downregulated DEGs, GO enrichment analysis was performed. The biological function of DEGs were assigned to biological process (BP), molecular function (MF) and cellular component (CC). The top enriched GO terms in BP group include metabolic processes, cell proliferation, cell cycle, cell cycle checkpoint, programmed cell death, cell differentiation, response to hypoxia, biosynthetic processes, nucleic acid metabolic pathways and cellular response to stress. Similarly, the GO analysis of MF showed that the DEGs were significantly enriched in carbohydrate derivative binding, nucleic acid binding, enzyme binding, kinase activity, transcription factor binding, glycoprotein binding, chromatin binding, cytoskeletal protein binding, purine ribonucleotide binding and cyclin-dependent protein serine/threonine kinase activity. The cellular component of GO analysis showed that majority of DEGs were enriched in cell, cytoplasm, nucleus, cytoplasmic part, microtubule cytoskeleton, chromosome, adherens junction, organelle part and extracellular region. Overall, the KEGG pathway and GO enrichment analysis of downregulated DEGs indicated that major pathways and cellular processes associated with tumorigenesis and metastasis are regulated by *MYCN*.

During the tumorigenic process, neoplastic cells rewire their metabolism and energy production to support rapid proliferation, accelerated biosynthesis, continuous growth, tolerance to adverse conditions, invasion and metastasis. Cancer cells preferentially depend on aerobic glycolysis even under normoxic condition, a phenomenon, known as Warburg effect [43]. Despite increasing evidence on the importance of tumor cell metabolism for cancer progression, only a few studies showed the role of *MYCN* in the regulation of metabolic processes in RB. Based on our microarray data, we specifically considered two hallmarks of cancer; altered metabolism and cell migration in RB in response to *MYCN* downregulation. The microarray data revealed that the key metabolic genes encoding enzymes, ENO2, HK2, TPI1, PDK1, ALDOC, PFKFB3, PFKFB4, LDHA, PGK1, PKM, PGAM1 and PGAM4 are among some of the top downregulated DEGs following *MYCN* knockdown. Further, the mRNA expression of the above-mentioned genes was validated using qRT-PCR and similar results were obtained. Previously, it was demonstrated that c-MYC is the key regulator of genes involved in glycolysis suggesting its role in metabolic switch to glycolysis during cell proliferation and tumorigenesis [44,45]. In a recent study, we have reported that PDK1, a key gate-keeping enzyme of glycolysis is overexpressed in RB and its promoter consists of *MYCN* binding motifs suggesting its possible regulation by *MYCN* [29,46]. Additionally, the dependency of *MYCN*-driven glioblastoma cells on glycolysis has been reported by Tateishi et al., 2016 [47]. However, numerous studies have provided additional insights into the metabolic reprograming in cancer cells. Vyas et al., 2016 and Viale et al., 2015 have described that cancer cells are highly dependent on oxidative phosphorylation preferably than glycolysis [48,49]. As our data hint at *MYCN* mediated regulation of glucose metabolism in RB, we studied the functional role of *MYCN* on additional metabolic parameters such as glucose uptake, lactate and ROS levels. Consistent with the microarray data, decreased glucose uptake, lactate and ROS levels were observed after *MYCN* inhibition in RB cells compared to the control cells. Taken together, our data suggest that *MYCN* regulates the key enzymes involved in glucose metabolism in rapidly proliferating RB cells. This finding is particularly interesting as retina is known to utilize glycolysis for its energy needs. However, further increase in expression of genes involved in glycolysis in RB as well as increased glucose uptake suggests that RB cells may utilize elevated glycolysis for their increased demand for energy production and synthesis of biosynthetic precursors required for rapid proliferation.

To decipher the role of *MYCN* in invasion and metastasis, we determined the mRNA expression of the genes that encode ECM proteins such as FN1, FBN1 and MMP9, and VEGFA. Decreased mRNA expression was observed in response to *MYCN* inhibition and that correlated with the microarray data. In addition, gelatin zymography showed a decreased gelatinolytic activity of active-MMP9 upon *MYCN* inhibition in RB cells. Further, wound healing assay revealed that the migratory ability of RB cells was diminished in *MYCN* depleted RB cells compared to the control cells. FN1 has been recognized to support cell proliferation and migration in multiple tumor types such as gastric cancer, colorectal cancer, thyroid cancer and esophageal squamous cell carcinoma [50,51,52,53]. Similarly, FBN1 has been reported to promote ovarian cancer metastasis [54,55]. MMP9 has been recognized as a possible biomarker for metastasis in various cancers such as ovarian cancer [56], non-small cell lung cancer [57], colorectal cancer [58] and breast cancer [59]. Recently, Webb et al., 2017 have shown that MMP2 and MMP9 drive metastatic pathways by promoting cell migration and viability and by releasing angiogenic factors in RB cells [60]. In agreement with the existing literature, our data suggest that *MYCN* promotes RB cell growth and metastasis possibly via regulating the genes involved in the ECM modification. *MYCN* in addition to the regulation of genes involved in glucose metabolism and migration, has been shown to positively modulate the expression of mitotic protein kinase, aurora kinase B in RB [30] and other tumors [61]. Overall, our data along with the published literature show that *MYCN* may regulate a plethora of cellular functions in RB and other tumors [62].

Based on the inhibitor and shRNA experiments, we further studied if the efficacy of chemotherapeutic agent, carboplatin could be enhanced in combination with MYC inhibitors. We treated RB cells with various combinations of carboplatin and small molecule inhibitors of *MYCN* and the results showed an increased inhibitory effect of combination treatment compared to the single treatment suggesting synergistic interaction. These results indicate the possibility of inhibiting *MYCN* in RB in combination with chemotherapy drug currently used in the clinic. Similarly, a recent study by Aubry et al., 2020 revealed that combination inhibition of RAD51 and topotecan synergistically inhibited RB cell growth and the cells resistant to this combination could be inhibited by further combining Navitoclax, a BCL2 inhibitor with RAD51 inhibition and topotecan [63]. Another interesting study showed that the combination treatment of cells that were RB1 wildtype with *MYCN* amplification and chemoresistant with Panobinostat and bortezomib or carboplatin inhibited tumor cell growth [5]. It would be interesting to see if *MYCN* inhibition could be combined with any of the other combination strategies proposed earlier for RB. Altogether, our study demonstrates that *MYCN* promotes cell survival and migration of RB cells possibly via regulating the key metabolic genes to provide the enhanced demand for biosynthetic substrates and energy production of the rapidly proliferating RB cells. In addition, *MYCN* also regulates the expression of the genes involved in the ECM modification to further drive RB progression and metastasis. However, additional studies are required to understand the role of individual genes in these *MYCN* mediated processes.

## 5. Conclusions

In the current study, we have verified the expression of *MYCN*, evaluated its inhibition as a potential therapeutic strategy and identified critical pathways downstream of *MYCN* in RB. Our study suggests that *MYCN* regulates RB cell growth and migration possibly via modulating the key genes involved in glucose metabolism and extracellular matrix modification and targeting it could be a potential therapeutic strategy. We also demonstrated the possibility of targeting *MYCN* using primary cells derived from enucleated eyes. This is of significance due to lack of suitable animal models for RB. The microarray analysis identified pathways that could be potential drug targets for RB. Our drug combination experiments provide rationale for developing additional therapeutic strategies for RB. Further studies are necessary to clarify the role of other critical pathways regulated by *MYCN*. Likewise, combination studies comprising of existing chemotherapy agents and additional *MYCN* inhibition strategies need to be evaluated.

## Figures and Tables

**Table 1:** Correlation analysis of *MYCN* expression with demographic and histological characteristics of RB patients.

Characteristic		Total	Positive	Negative	Statistical Test	*p*-Value	Remarks
Age at diagnosis (Months)	Median		36	24	Student’s *t* test	0.42	NS
	Mean		33.61 ± 24.30	28.90 ± 16.41			
	Range		1–96	12–84			
Gender	Male	37	24	13	Chi-square test	0.56	NS
	Female	21	12	9			
Laterality	Unilateral	52	31	21	Chi-square test	0.25	NS
	Bilateral	6	5	1			
Chemotherapy	Yes	16	8	8	Chi-square test	0.24	NS
	No	42	28	14			
Choroid invasion	Present	28	18	10	Chi-square test	0.73	NS
	Absent	30	18	12			
Optic nerve invasion	Present	34	18	16	Chi-square test	0.08	NS
	Absent	24	18	6			
Anterior chamber invasion	Present	9	4	5	Chi-square test	0.23	NS
	Absent	49	32	17			
Scleral invasion	Present	9	5	4	Chi-square test	0.66	NS
	Absent	49	31	18			
Differentiation	Well	7	4	3	Chi-square test	0.95	NS
	Moderate	11	7	4			
	Poor	34	21	13			
	Undifferentiated	6	4	2			

NS: Not significant.

## Data Availability

The microarray data for *MYCN* scrambled and knockdown cells was submitted in the repository of “Gene Expression Omnibus” with the accession number GSE168903, https://www.ncbi.nlm.nih.gov/geo/query/acc.cgi?acc=GSE168903, accessed on 14 October 2021.

## Supplementary Materials

The following are available online at https://www.mdpi.com/article/10.3390/cancers13205248/s1, Table S1: IC50 concentrations for small molecule inhibitors of *MYCN* in RB cells, Figure S1: Inhibition of *MYCN* has no adverse effect on the cell viability of untransformed human retinal pigment epithelial (ARPE-19) cells, Figure S2: Inhibition of *MYCN* induces accumulation of cells in G0/G1 phase of the cell cycle and increases apoptosis in RB cells, Figure S3: Identification and enrichment analysis of differentially expressed genes in *MYCN* knockdown Y79 cells, Table S2: Primers used for qRT-PCR validation of identified target genes, Figure S4: qRT-PCR validation of identified downregulated metabolic genes, Figure S5: Dose response curves for *MYCN* inhibitors (JQ1 and 10058-F4), carboplatin and combination of carboplatin with JQ1 or 10058-F4, and Figure S6: Full immunoblots for Figure 1A, Figure 3C and Figure 4A.
