# Supplementary material for: The Oncogene MYCN Modulates Glycolytic and Invasive Genes to Enhance Cell Viability and Migration in Human Retinoblastoma
Source: Cancers (Basel). 2021 Oct 19;13(20):5248. doi: 10.3390/cancers13205248 (PMC8533785; doi:10.3390/cancers13205248)
Supplement: Supplementary file 1 [file cancers-13-05248-s001.zip › cancers-1395127-supplementary.pdf]

*Supplementary Material*

# The oncogene MYCN modulates glycolytic and invasive genes to enhance cell viability and migration in human retinoblastoma

Swatishree Sradhanjali, Padmalochan Rout, Devjyoti Tripathy, Swathi Kaliki, Suryasnata Rath, Rahul Modak, Ruchi Mittal, Tirumala K. Chowdary and Mamatha M. Reddy

**Table S1.** IC50 concentrations for small molecule inhibitors of MYCN in RB cells

| Drugs | IC50 values | | | |
|---|---|---|---|---|
| | Y79 | Weri-Rb1 | LRB1 | LRB2 |
| JQ1 (nM) | 83.75 | 26.91 | 134.89 | 112.20 |
| I-BET762 (nM) | 114.21 | 16.21 | 144.54 | 263.02 |
| 10058-F4 (μM) | 16.98 | 12.88 | 12.58 | 12.02 |
| 10074-G5 (μM) | 13.80 | 7.94 | 11.48 | 8.91 |

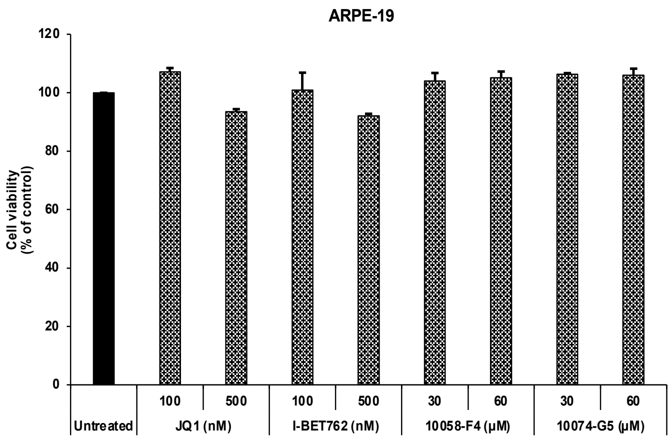


**Figure S1.** Inhibition of MYCN has no adverse effect on the cell viability of untransformed human retinal pigment epithelial (ARPE-19) cells.

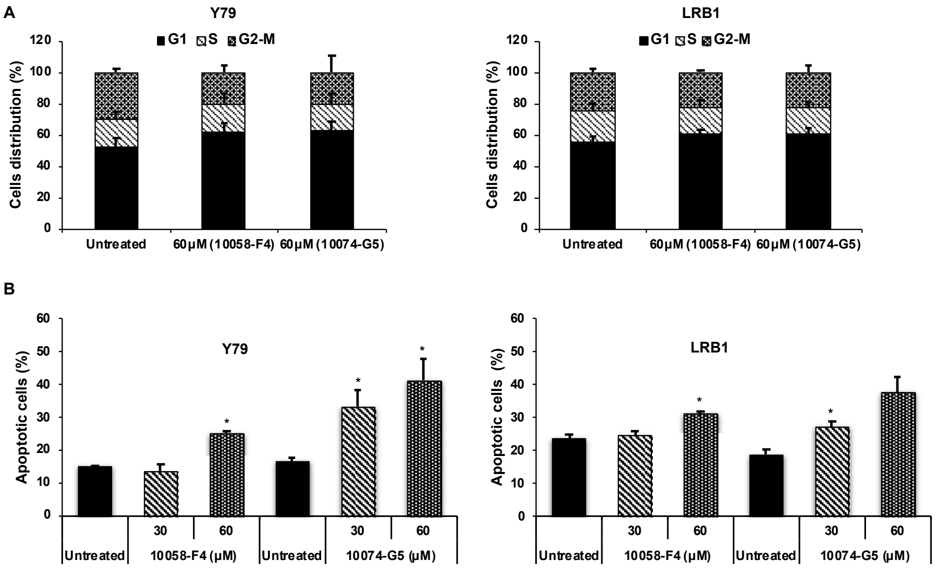


**Figure S2.** Inhibition of MYCN induces accumulation of cells in G0/G1 phase of the cell cycle and increases apoptosis in RB cells. Y79 and LRB1 cells were treated with 10058-F4 and 10074-G5. (**A**) Cell cycle was analyzed by flow cytometry. (**B**) Percent apoptotic cells in inhibitor treated Y79 and LRB1 cells. Error bars represents standard error of mean (* $p<0.05$ vs untreated).

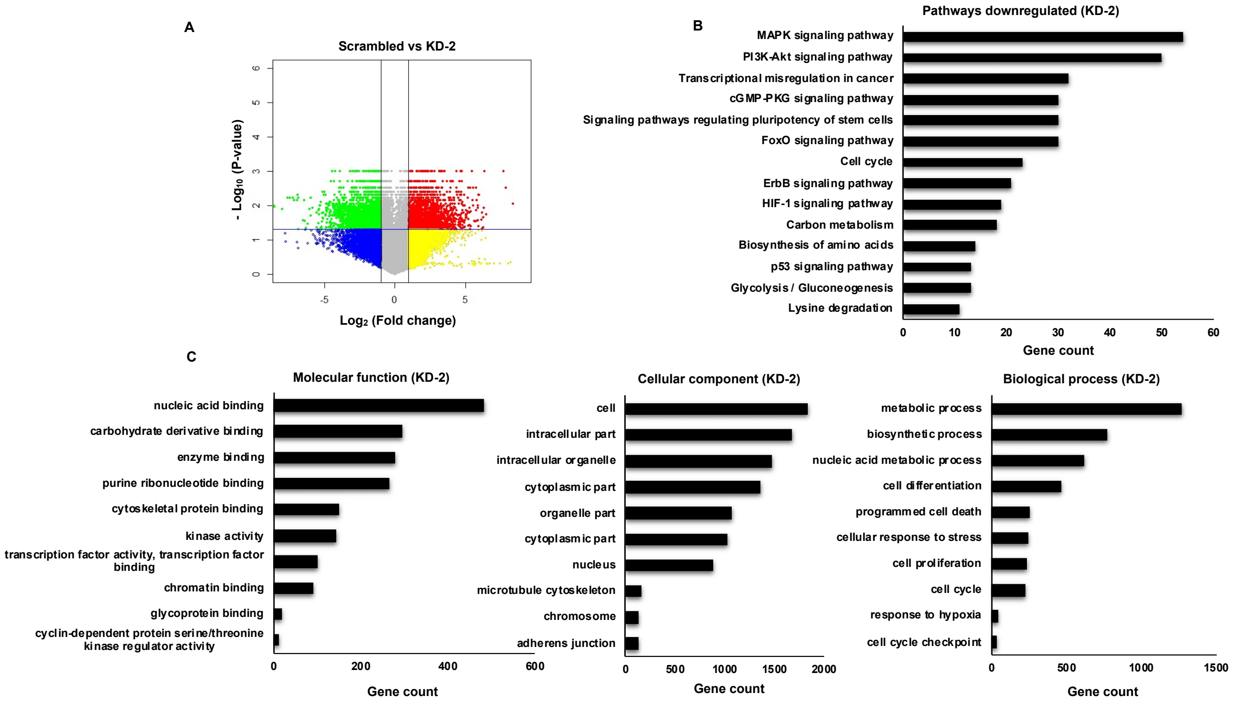


**Figure S3.** Identification and enrichment analysis of differentially expressed genes in MYCN knockdown Y79 cells. (**A**) Volcano plot representing the differentially expressed genes upon MYCN knockdown (KD-2). The green dots indicate significantly downregulated genes with log2 (fold change) < -1 and p-value < 0.05, and red dots represent the significantly upregulated genes with log2 (fold change) > 1 and p-value < 0.05. (**B**) Top 14 enriched pathways identified using KEGG pathway analysis of downregulated DEGs. (**C**) Statistically enriched functions identified using GO functional enrichment analysis. The functions are classified as molecular function, biological process and cellular component group. KEGG: Kyoto Encyclopedia of Genes and Genomes, GO: Gene Ontology, DEGs: Differentially Expressed Genes.

**Table S2.** Primers used for qRT-PCR validation of identified target genes

| Target | Forward primer (3'-5') | Reverse primer (3'-5') |
|---|---|---|
| ENO2 | TGTCTCATCCTCCTGGAACC | TCAATCAGGGAAGTTCTGGG |
| HK2 | AACCATGACCAAGTGCAGAA | AGCCCTTTCTCCATCTCCTT |
| TPI1 | CACTGAGAAGGTTGTTTTCG | TAAATGATACGGGTGCTCTG |
| PDK1 | CAACAGAGGTGTTTACCCCC | ATTTTCCTCAAAGGAACGCC |
| ALDOC | CAGGGCAATGTCAGACAACT | GGCTGCGGCTGCTAACT |
| PFKFB3 | CAGTTGTGGCCTCCAATATC | GGCTTCATAGCAACTGATCC |
| PFKFB4 | GGAGTTCAATGTTGGCCAGT | TCAGGATCCACACAGATGGA |
| LDHA | AGCCCGATTCCGTTACCT | CACCAGCAACATTCATTCCA |
| PGK1 | GGGAAAAGATGCTTCTGGGAA | TTGGAAAGTGAAGCTCGGAAA |
| PKM | ATCGTCCTCACCAAGTCTGG | GAAGATGCCACGGTACAGGT |
| PGAM1 | ATGATGTCCCACCACCTCCGAT | ATCCTTCAGACTCTCACAGGAG |
| PGAM4 | CTAAGCCACGACCAATGAG | CAGAAGAGCAGAGGACAGAC |
| MYCN | TCGCAGAAACCACAACAT CC | CAAGTCCGAGCGTGTTC AAT |
| β2-M | TATCCAGCGTACTCCAAAGA | GACAAGTCTGAATGCTCCAC |
| MMP9 | GCCACTACTGTGCCTTTGAGTC | CCCTCAGAGAATCGCCAGTACT |
| VEGF | AGCTGCGCTGATAGACATCC | CTACCTCCACCATGCCAAGT |
| FN1 | AAGGTTCGGGAAGAGGTTGT | GGGAAACTGTGTAGGGGTCA |
| FBN1 | TGACTGGCCCACACGTGCATAG | TGACATTGACCCCTTGTTGACAGGA |

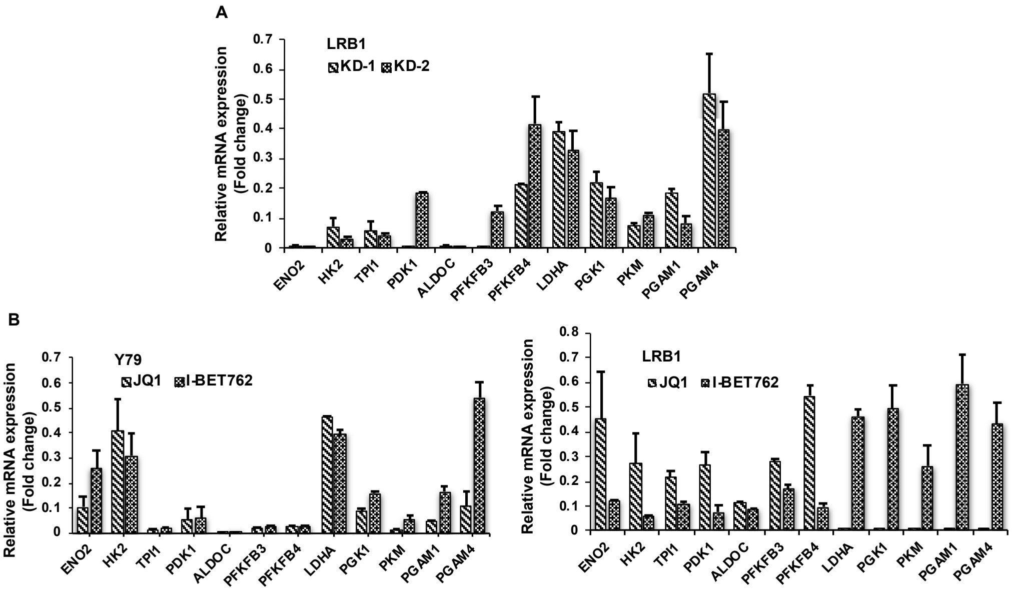


**Figure S4.** qRT-PCR validation of identified downregulated metabolic genes. (**A**) MYCN knockdown in LRB1 cells resulted in decreased mRNA expression of the key metabolic genes identified by microarray. (**B**) Y79 and LRB1 cells treated with small molecule inhibitors of MYCN showed a decreased mRNA expression level of metabolic genes similar to microarray data. The error bars represent the standard error of mean.

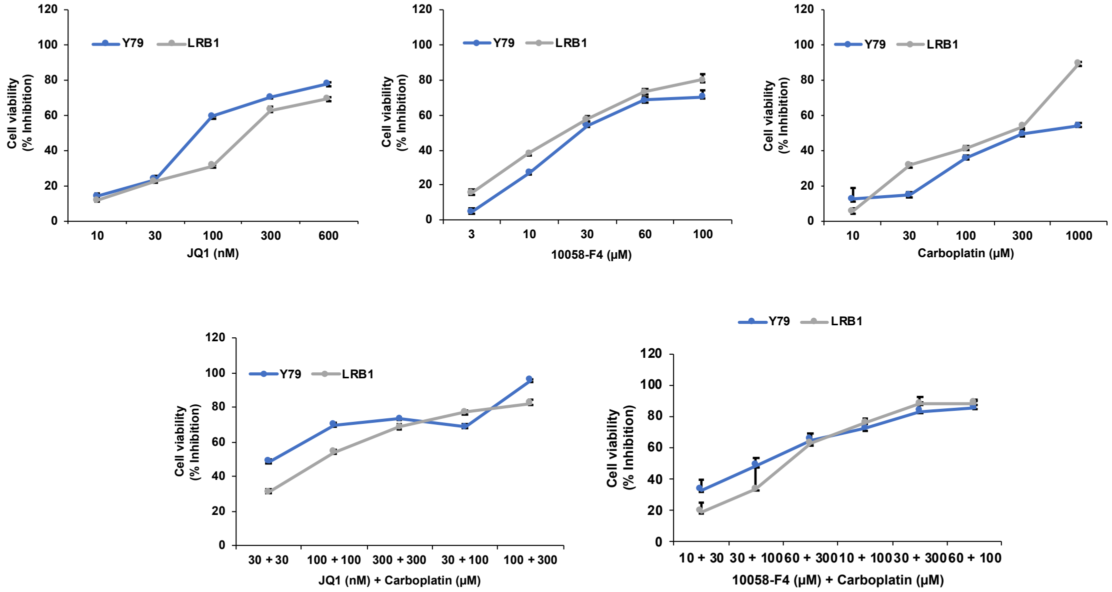


**Figure S5.** Dose response curves for MYCN inhibitors (JQ1 and 10058-F4), carboplatin and combination of carboplatin with JQ1 or 10058-F4.

**Figure S6: Original immunoblots**

**Full western blots for Figure 1A**

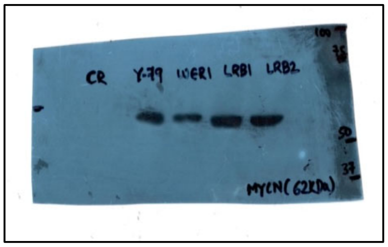


MYCN (63 kDa)

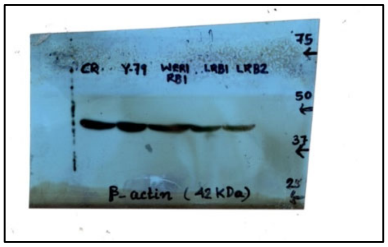


β-actin (42 kDa)

Figure 1A Top panel

Figure 1A- Bottom panel

**Full western blots for Figure 3C**

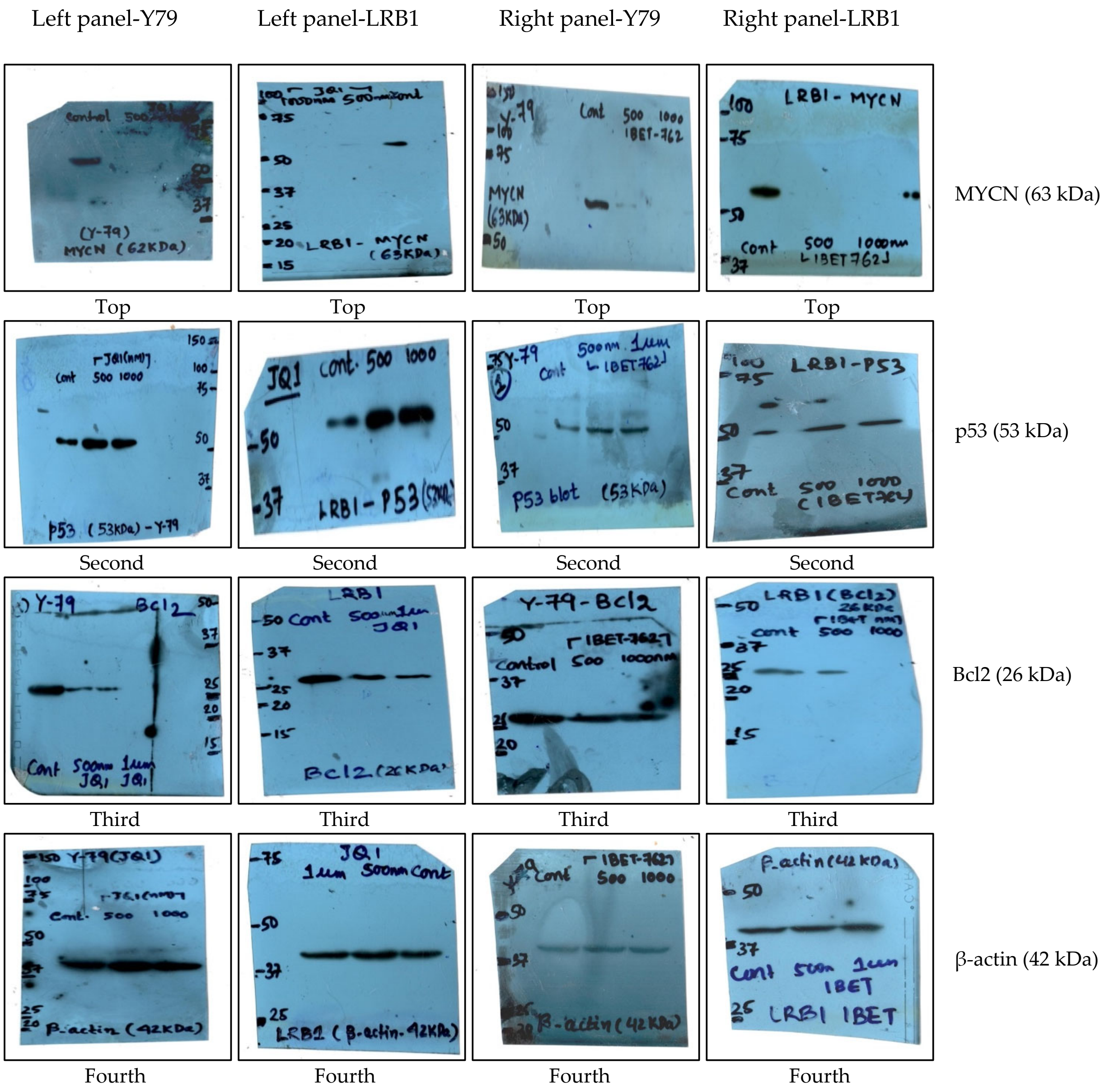

**Full western blots for Figure 4A**

Left panel- Y79 Right panel- LRB1

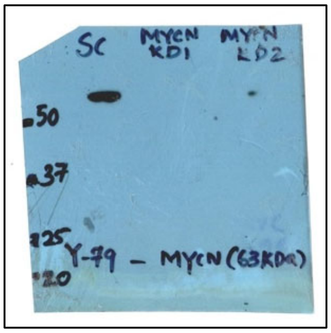

Top

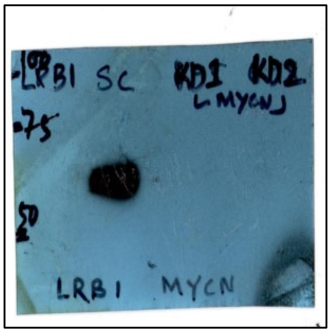

Top

MYCN (63 kDa)

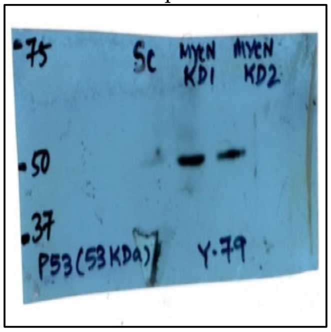

Second

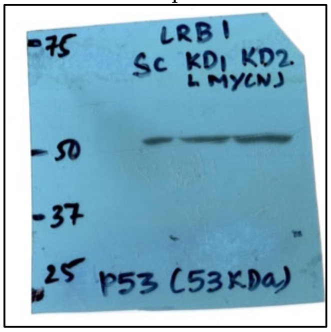

Second

p53 (53 kDa)

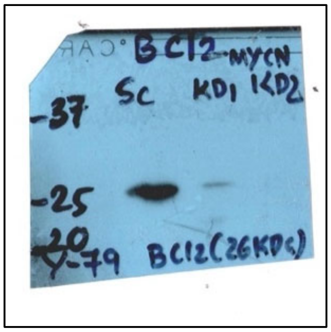

Third

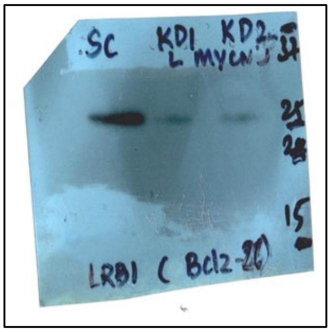

Third

Bcl2 (26 kDa)

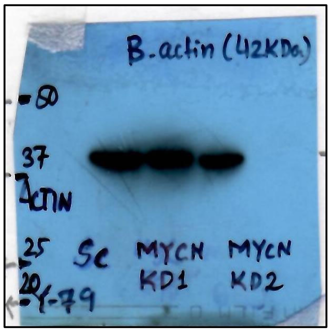

Fourth

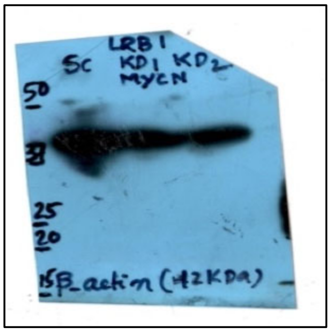

Fourth

β-actin (42 kDa)
